# The Nanosystems Involved in Treating Lung Cancer

**DOI:** 10.3390/life11070682

**Published:** 2021-07-13

**Authors:** Andreea Crintea, Alina Gabriela Dutu, Gabriel Samasca, Ioan Alexandru Florian, Iulia Lupan, Alexandra Marioara Craciun

**Affiliations:** 1 Department of Biochemistry, “Iuliu Hatieganu” University of Medicine and Pharmacy, 3400 Cluj-Napoca, Romania; crintea.andreea@umfcluj.ro (A.C.); Alina.Dutu@umfcluj.ro (A.G.D.); acraciun@umfcluj.ro (A.M.C.); 2 Department of Immunology, “Iuliu Hatieganu” University of Medicine and Pharmacy, 3400 Cluj-Napoca, Romania; 3 Department of Neurosciences, “Iuliu Hatieganu” University of Medicine and Pharmacy, 3400 Cluj-Napoca, Romania; florian.ioan@umfcluj.ro; 4 Interdisciplinary Institute of BioNanoScience, 3400 Cluj-Napoca, Romania; iulia.lupan@ubb.cluj.ro

**Keywords:** lung cancer, nanoparticles, nanosystems, liposomes, dendrimers, polymers, micelles, inorganic nanoparticles, siRNA delivery systems, biocompatibility

## Abstract

Even though there are various types of cancer, this pathology as a whole is considered the principal cause of death worldwide. Lung cancer is known as a heterogeneous condition, and it is apparent that genome modification presents a significant role in the occurrence of this disorder. There are conventional procedures that can be utilized against diverse cancer types, such as chemotherapy or radiotherapy, but they are hampered by the numerous side effects. Owing to the many adverse events observed in these therapies, it is imperative to continuously develop new and improved strategies for managing individuals with cancer. Nanomedicine plays an important role in establishing new methods for detecting chromosomal rearrangements and mutations for targeted chemotherapeutics or the local delivery of drugs via different types of nano-particle carriers to the lungs or other organs or areas of interest. Because of the complex signaling pathways involved in developing different types of cancer, the need to discover new methods for prevention and detection is crucial in producing gene delivery materials that exhibit the desired roles. Scientists have confirmed that nanotechnology-based procedures are more effective than conventional chemotherapy or radiotherapy, with minor side effects. Several nanoparticles, nanomaterials, and nanosystems have been studied, including liposomes, dendrimers, polymers, micelles, inorganic nanoparticles, such as gold nanoparticles or carbon nanotubes, and even siRNA delivery systems. The cytotoxicity of such nanosystems is a debatable concern, and nanotechnology-based delivery systems must be improved to increase the bioavailability, biocompatibility, and safety profiles, since these nanosystems boast a remarkable potential in many biomedical applications, including anti-tumor activity or gene therapy. In this review, the nanosystems involved in treating lung cancer and its associated challenges are discussed.

## 1. Introduction

Cancer, in general, is a major cause of mortality on a global scale. This intricate pathology is characterized by inherent genetic alterations and cellular disorders, denoting an abnormal and uncontrollable cellular growth, eventually leading to the death of the patient [1,2,3].

According to the World Health Organization, Europe accounts for 23.4% of global cancer cases and 20.3% of cancer deaths [4,5]. Lung, breast, and colorectum cancer are the top five cancer types in terms of mortality. The majority of the newly discovered cases are related to lung and breast cancer [6]. Lung cancer is responsible for the most significant number of deaths out of all cancer types due to the treatment difficulties and the poor prognosis on a worldwide scale, also representing the leading cause of cancer death in men. Lung cancer can be classified into two categories, depending on the cell morphology [7,8]. Non-small cell lung carcinoma (NSCLC) is considered an aggressive type of cancer, but NSCLC itself pales in comparison to small cell lung carcinoma (SCLC) in terms of aggressiveness [1,3,5,9]. 

Tumors can either be removed from the body, even during their early stages of development, or treated via no-invasive methods [10]. For example, lung cancer can be managed by various fundamental methods, each with its own set of limitations: for one, surgery cannot always lead to complete removal, whereas radiation therapy may cause a reduction of tumor size, but it too will never lead to complete eradication, and photodynamic therapy or chemotherapy represent different methods that can be employed for advanced-stage lung cancer [11,12,13]. Moreover, radiation therapy and chemotherapy indiscriminately affect the cancerous cells, as well as the healthy tissues [14,15]. Another crucial aspect that should be taken into consideration is that radiation therapy and chemotherapy have various side effects, such as anemia, neutropenia, nausea, diarrhea, and other gastrointestinal symptoms, and to reduce the severity and frequency of these events requires the intake of additional drugs [16]. The typically late diagnosis and the standard treatments, which are characterized by many side effects and a lack of personalized therapy contribute to the high mortality, factors reinforcing the necessity to develop a new approach for this condition [17,18,19].

## 2. Nanoparticles—Characterization and Classification

Nanoparticles and nanostructured materials have an important role in nano-biomedical technology due to their characteristics and multiple application domains [20,21]. According to the British Standards Institution, nanoparticles are defined as: “Nano-objects with three external nanoscale dimensions. The terms nanorod or nanoplate are employed, instead of nanoparticle when the longest and the shortest axis lengths of a nano-object are different.” Nanostructured materials are defined as: “Materials containing an internal or surface nanostructure” [22,23,24]. Nanoparticles are stable, colloidal particles ranging in size between 1–100 nm, and their properties dictate their behavior in vivo [21,22]. 

The morphological features of the nanoparticles can affect their circulation and target inside the body [25]. Using different techniques, more and more nanoparticles are being produced and are responsible for targeting specific cell signaling [26]. Table 1 illustrates the characteristics and applications of the nanosystems currently in use for lung cancer.

The most vital aspects of nanotechnology are the development of a proper synthesizing method that can reduce toxicity, surface modifications, and the therapeutic design of nanoparticle-based formulations in cancer, because these agents possess a crucial use as both therapeutic and diagnostic tools [41,42]. Nanoparticles can be categorized according to their size, morphology, and surface charge using advanced microscopic techniques and are primarily characterized by particle size distribution and morphology [43,44,45]. Notably, the size of the nanoparticles has a fundamental effect on the drug release—they possess a larger surface to mass ratio than other compounds, meaning an improved capacity to bind, absorb, and carry therapeutic agents [46,47]. Owing to their small size, geometry, and large surface area, drug nanoparticles can also cross the blood–brain barrier, and their capacity to enter the pulmonary system or to be absorbed is very high [48,49].

Nanomaterials can be utilized as delivery tools by encapsulating drugs or associating therapeutic drugs and distributed to target tissues accurately with a controlled release [50]. Surface modification with poly(ethylene glycol) may lead to an increased presence in the circulation by avoiding recognition and phagocytosis by the mononuclear phagocytic system. The purposes for the nanoparticle entrapment of medications are either an improved delivery and uptake by cells and/or a reduction in toxicity. Chemical features, such as surface charge, may decide the fate of nanoparticles in cells [51]. Surface modifications of nanoparticles allow for medical opportunities, such as drug targeting in terms of cellular adhesion and invasion and transcellular transport. Coupling distinct proteins, such as antibodies, to the nanoparticle surface may allow for a more specific immune-directed targeting of the particles to certain cells or organs [52]. The use of nanoparticles as drug carriers may reduce the toxicity of the incorporated drug, although the distinction between the drug and the nanoparticle toxicity cannot always be made, and many problems still need to be solved regarding cancer treatment, diagnostics, and imaging [53].

There is a distinctive mechanism that is responsible for the distribution of the nanoparticles [54,55]. After nanoparticles enter the human body via systemic circulation, particle–protein interaction ensues [56]. The lymphatic system has a dual responsibility regarding nanoparticles, both delivering them toward and discarding them from target tissues. It has been shown that microparticles larger than 7 µm are filtered mechanically, the reticuloendothelial system is capable to detect particles with diameters between 0.1–7 µm in the liver or spleen, and particles with a diameter lower than 100 µm will remain in the blood vessels until macrophages clear them from the body [47,57,58]. The main advantage of nanotechnology for cancer treatment is associated with tumor-targeting, which implies the capability of differentiating malignant cells from nonmalignant cells and ultimately eradicating the tumoral cells [59]. There are two processes involved in understanding malignant cells and nonmalignant cells with designed nanocarriers via active and passive targeting [47,57,60].

**Figure 1:**
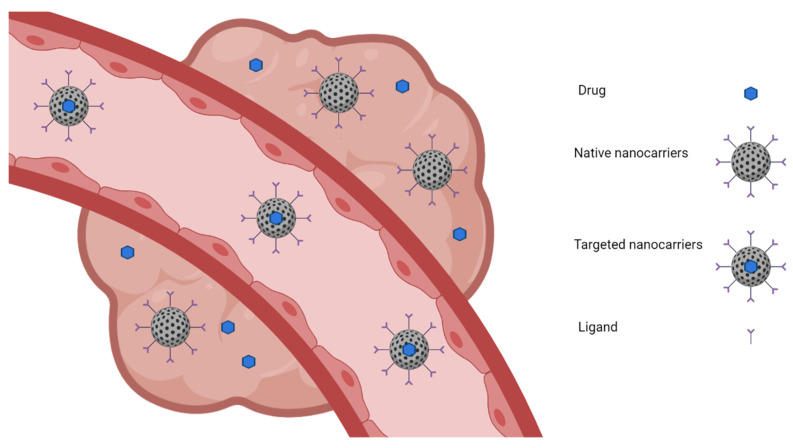
Passive and active targeting.

As exemplified in Figure 1, passive targeting is achieved when specific drugs, especially chemotherapeutic agents, are loaded into a native nanocarrier that passively reaches the solid tumor [61,62]. In the case of tumors, this type of targeting takes advantage of hyperpermeable cells and impaired lymphatic drainage [63]. Nanoparticles begin to accumulate inside the tumors due to their ineffective lymphatic drainage, and an important fact that should be taken into consideration, in this case, is that the size of the nanoparticles should be less than 200 nm, and their surface must be hydrophobic to prevent clearance by macrophages [64,65,66]. On the other hand, during active targeting, a particular drug is loaded into a native nanocarrier, and the nanocarrier system is loaded into a tissue-specific targeting ligand or a cell-specific targeting ligand [67,68]. In the active targeting, the nanocarriers’ surface can be modified with ligands that are recognized by the cell’s receptors. The content of the nanoparticles can be released in proximity to the target cell, attached to the membrane of the specific cell, or internalized in the cell [69,70]. 

## 3. Nanosystems Involved in Treating Lung Cancer

Even if the early symptoms of lung cancer may be frequently overlooked, and the late stages of this condition could become inoperable, there are still just two main cancer drug therapies based on nanotechnology approved by the Food and Drug Administration: Abraxane and Genexol-PM [71].

Used in several types of cancer (such as breast, pancreatic, or non-small-cell lung cancer), Abraxane mainly consists of paclitaxel bound by albumin in the form of nanoparticles. Considered alone or combined in chemotherapy, this medicine proved to be effective as part of the lung cancer cure, showing milder adverse effects and great tolerability when administered alone [72] as a chemotherapeutic agent or in conjunction with other traditional drugs, such as cisplatin [73] or carboplatin [74].

With the same active substance (paclitaxel), Genexol-PM is the second drug approved by FDA for usage in lung cancer treatment. The main difference between it and Abraxane is the nanocarrier, which, in the case of Genexol-PM, consists of a proprietary polymeric micelle technology, according to the producer’s website. Unlike studies on Abraxane administration, the use of paclitaxel in the form of Genexol-PM seems to be more controversial. Even if there is clear evidence of a superior tolerance in comparison with plain paclitaxel administration [75], and studies show its remarkable efficacy in treating lung cancer [76,77], at least one study [78] highlighted that serious safety concerns need to be assessed in the future. Nevertheless, while phase III clinical studies are still ongoing, back in 2013, Genexol-PM was regarded as the most successful micellar formulation of paclitaxel [79], and considering there have been no other related FDA approvals up to the present, this statement should still be valid.

These two alternatives based on nanosystems available in the USA for the cure of lung cancer reflect the very beginning point where we are at the moment and also the fact that there is a great amount of research that still needs to be done in order to achieve new milestones in this direction. One of the most promising solutions would be the development of immunotherapies. Already becoming a notable emerging domain, it can be conveniently used in the form of novel formulations, such as combinations of drug-loaded nanoparticles and immune checkpoint inhibitors (ICIs) [80]. An elegant and encouraging solution to this issue was proposed by Ge and collaborators [81], in which Fe_3_O_4_ superparticles (SPs) would encapsulate and carry immune-adjuvant drugs to a magnetic-targeted site. Using complementary photothermal therapy (PTT) under near-infrared laser irradiation, this method could lead to both direct and indirect ways (*via* immune system activation) to significantly reduce the tumor volume.

Finally, with the drug resistance of tumors still being a major problem, one’s genetic traits and the ability of physicians to address this issue remain important decisive factors [82]. Gene therapy came in response to this specific problem and offered a wide range of solutions, from the use of silencing (si) RNA or long-non-coding (lnc)RNA to avoid the synthesis of pro-tumoral proteins, to micro RNA (miRNA) administration for gene expression modulation or even the novel CRISPR/Cas9 system for very specific gene targeting [83], all of which may be, at any time, promising candidates for lung cancer therapy.

Conclusively, even if there is a wide range of possibilities available for lung cancer therapy development, the actual results are rather modest, and the entire process seems to be evolving heavily at the moment. The intra- and inter-individual heterogeneity of this disease, corroborated by the increased instability or low encapsulation efficiency of the nanocarriers and other safety-related issues mentioned above, remain important concerns that must be addressed in the future. 

### 3.1. Organic Nanosystems

#### 3.1.1. Lipid-Based Particles

Liposomes are distinguished by their unique structure, represented by the lipid bilayer. This lipid-based vesicle is similar to cellular membranes, has an augmented biocompatibility like other synthetic materials, and has the potential to be a useful drug vehicle, as it is intended to be a nanocarrier [84,85]. The research is focused on their utilization as nanocarriers of drugs with a high toxicity, such as those employed in oncology. Under these circumstances, liposomes can present a great advantage in terms of permitting the transport of specific agents and allowing for a controlled release of the drug within a particular organ [32,63,67]. Another advantage of using liposomes in therapy is that they protect the loaded drug from degradation and prevent undesirable exposure to the environment [86].

Liposomes can be classified according to their size, the number of bilayers, or the preparation method: multilamellar vesicles that consist of several lipid bilayers separated from one another by aqueous spaces, which are heterogenous in size: small unilamellar vesicles comprised of a single bilayer surrounding the entrapped aqueous space, possessing a diameter less than 100 nm; or large unilamellar vesicles composed of a single bilayer surrounding the entrapped aqueous space, with a diameter larger than 100 nm [83,87].

The release of the drug can be deliberately triggered by different techniques, such as ultrasound, light, magnetism, or hyperthermia. Several experts in the field attempted to modify the surface of the liposomes to improve their capability to target different types of cancer and accumulate at the site of the tumors, delivering a higher concentration of the drug [32,88,89,90]. Liposomes can also be employed to alter DNA, anticancer agents, and antibiotics to improve chemotherapy by adding specific molecules to their surface, according to the tumor type or gene delivery, these being the most encouraging tools for cancer gene therapy [91,92,93]. Currently, there are only two products available on the market that can be utilized for ovarian cancer and lymphoblastic leukemia [94]. 

Regarding liposome usage in lung cancer treatment, a specific and outstanding benefit noticed was the uniform particle size distribution with respect to liposome, operating as drug delivery agents. There are at least a few studies in which the biodistribution of these formulations was indicated as an evidently strong point for choosing them as medication carriers [95].

#### 3.1.2. Polymer-Based Particles

Dendrimers are a unique class of highly branched macromolecules whose shape and size can be controlled. These polymetric molecules are made up of multiple branched monomers capable of self-organization [29,96]. Structurally, the dendrimers are constituted by three essential regions: a central core, branches, or end groups, and the surface is formed using convergent or divergent step-growth polymerization, starting from monomers [97]. The size of these polymeric nanostructures depends on the number of branching points, which can be controlled and begin from a spherical central core. The cavities shaped inside the core structure and folds of the branches form cages and channels [98]. The free ends of the dendrimer arrangement can be used to attach other molecules, such as liposomes, nanoparticles, carbon nanotubes, anticancer compounds, or radioligands, or they can be transformed into biocompatible compounds with a high bio-permeability and low cytotoxicity [99,100]. Dendrimers present a variety of qualities, such as a surface functionalization capability and monodispersity of size, which make them attractive candidates for gene therapy—due to their ability to enter the cells via endocytosis—or for drug delivery and anticancer therapy, including chemotherapy [101,102]. If we refer to dendrimers as nanocarriers for drug delivery, the specific drug molecules can be quickly included via ligand- or receptor-mediated endocytosis [96].

Dendrimers show many advantages, such as a high drug-loading capacity, nano-size, which is favorable for targeting, and the capability to improve the solubility of poorly soluble anti-neoplastic drugs [103,104]. Nevertheless, their intrinsic toxicity cannot be disregarded—all classes of dendrimers manifest cytotoxic and hemolytic characteristics. This toxicity is dependent on the specific features of dendrimers and is related to the surface end groups [102,105]. To minimize the toxicity, polyethylene glycol can be associated or conjugated, as it can improve the plasma circulation time and tumor accumulation through an enhanced permeability and retention [106]. Different varieties of dendrimers can be utilized for multiple purposes, such as drug-encapsulated dendrimers or dendrimer drug conjugates that boast several benefits over drug-encapsulated systems. These nanocarriers can pass through several delivery barriers using two distinct mechanisms: passive and active targeting [107].

Regarding lung cancer treatment management using dendrimers, several studies have already shown promising outcomes. Doxorubicin (DOX), Cis-diamminodichloridoplatinum (II) (CDDP), and cisplatin (cisPt) are just a few of the efficient anti-tumoral medications tested as loads for dendrimers that are worth mentioning [108].

Polymers can be divided into natural polymers, synthetic polymers, and microbial fermentation polymers, but only natural and synthetic ones can be used for nano delivery. Polymeric nanoparticles are solid, nanosized colloidal particles that consist of a biodegradable polymer that should be biocompatible and non-toxic [109,110,111]. These features are the most important when this nanoparticle is desired for use in drug delivery and gene therapy, as well as other applications. Natural polymers are obtained directly from natural resources, as opposed to synthetic polymers, which are modified or synthesized in the laboratory using different techniques and devices and are frequently used for nanoparticle design and development [32,64]. The most widely used polymer is chitosan, whereas other polymers are extensively used in nanoparticle synthesis, including dextran, albumin, heparin, gelatin, or collagen. Natural polymeric nanoparticles are biocompatible and non-toxic; however, when this type of nanoparticle is delivered across different biological membranes, issues such as on-site stability and a local variation in pH levels may sometimes limit their usefulness [64,65,66]. 

Synthetic polymers, such as polylactic acid, polyglycolic acid, and polyhydroxybutyrate, or other families of polymers are usually employed and suitable for drug delivery due to their individual characteristics, such as biocompatibility and biodegradability [112,113]. Synthetic polymeric nanoparticles present a particularly excellent result in terms of the release of drugs within the lungs in a controlled manner. They are a good candidate for oral, intravenous, or combined administering because of their advantages: biocompatibility and biodegradability, inferior toxicity, and low cost of production in large quantities using multiple methods [32,111]. Based on their structural organization, polymeric nanoparticles can be divided into nanocapsules and nanospheres. There have been numerous attempts to deliver a variety of anticancer drugs using polymeric nanoparticles, considering the physicochemical properties of polymers, their degradation, and the accurate and controllable drug release rate [32,114]. Moreover, it is also possible to synthesize polymeric nanoparticles with specific sizes, shapes, and surface modifications, offering a heightened precision in delivering a particular drug. All these developments have established a new direction in cancer treatment [115,116]. There is a large number of polymeric nanoparticles that have already been used in different phases of clinical trials—Abraxane has been approved by the Food and Drug Administration (FDA) for the treatment of different types of malignancies, such as breast cancer, NSCLC, and pancreatic cancer, or BIND-014, which is the first targeted polymeric nanoparticle utilized for the treatment of metastatic melanoma and squamous cell carcinoma [49,117,118].

Regarding nanocapsules, the drug is dissolved or dispersed in a liquid core of oil or water, which is encapsulated by a solid polymeric membrane, or in the case of the nanospheres, the drug is dispersed/entrapped in the polymer matrix. In both cases, the absorption or chemical conjugation of the drug on the surface is possible. As mentioned above, among the most important characteristics for polymers are biocompatibility and biodegradability; being biodegradable, these polymers can be degraded into individual monomers inside the body and removed from the body through metabolic pathways [32,40,48].

Micelles are nanosized, spherical colloidal particles, and lipid nanostructures consist of a hydrophobic core and a hydrophilic shell. In an aqueous environment, micelles hide their hydrophobic groups inside the structure and expose hydrophilic groups, whereas inside environments rich in lipids, these nanostructures are organized in the opposite way [119,120,121]. Micelles represent another variant of nanosystem that can be used to treat and diagnose multiple types of cancer and deliver various anticancer agents. By producing different variations of these nanosystems, it will be possible to monitor the pathways of interest and to estimate the therapeutic response [32,122,123]. Micelles are an innovative drug delivery system due to their stability in physiological conditions, high and versatile loading capacity, high accumulation of drugs at the target site, and their possibility of functionalizing the end group [38]. Medications can be entrapped within the hydrophobic core or linked covalently to the shell of these nanosystems. Micelles are stable and have a prolonged circulation time within the bloodstream, evading host defenses [124,125]. The nanocarriers’ ability to circumvent passive targeting via the fenestrated vasculature of tumors can be improved by covalent conjugation with the polyethylene glycol of the micelles’ surface. In an aqueous environment, the hydrophobic core of the micelles can solubilize water-insoluble drugs, and the shell of the micelles can adsorb polar molecules [38,39]. In contrast, drugs with an intermediate polarity can be distributed along with the surfactant molecules in intermediate positions. Many micelles that contain anticancer drugs are under clinical trials, and only one of these nanosystems is approved for treating breast cancer patients [124]. Specifically, with regard to cancer lung management, one of the greatest advantages posed by micelles are the facile methods used for modifying their surfaces and the great specificity shown by these adjusted particles for the lung tumor environment [126]. Docetaxel (DTXL), Paclitaxel, and cisPt in combination with etoposide (ETO) are some of the most important anti-tumoral drugs for which micelles served as nanocarriers in lung cancer treatment studies [127].

### 3.2. Inorganic Nanomaterials

Inorganic materials, such as gold, silver, silica, or platinum, are intensely used to produce metallic nanoparticles using different methods. The manufactured metallic nanoparticles present an organized three-dimensional arrangement [128,129]. They are more flexible than other types of nanoparticles because of the possibility of controlling their size, shape, structure, composition, assembly, or encapsulation. Even though metallic nanoparticles present several advantages, a series of shortcomings should be taken into consideration within specific biomedical applications, such as the impossibility of loading drugs into their structure, and the blood-related adverse effects and cytotoxicity, depending on their size, concentration, and time of exposure [21,24,86]. Of all metallic nanoparticles, gold nanoparticles are of great interest for biomedical applications and present an excellent efficiency against different types of cancer, low toxicity, and tunable optical properties that can be controlled and employed for the treatment and diagnosis of specific pathologies [24,130,131]. Gold nanoparticles are considered a suitable nanocarrier for the effective delivery of bioactive agents, drug delivery, or delivery of biomolecules, like proteins, DNA, and small interfering RNA (siRNA), bioassay detection or imaging [35,131]. The surface of gold nanoparticles can be functionalized with different ligands, such as peptides, proteins, or DNA. Gold nanoparticles are widely used in cancer therapy, including photothermal therapy, radiotherapy, or as angiogenesis inhibitions. The formation process of new blood vessels is also a remarkable opportunity for the use of gold nanoparticles in cancer therapy [47,132,133].

#### Non-Polymeric Particles

Gold nanoparticles are intensely studied in connection with lung cancer therapy and diagnosis. In combination with Methotrexate, gold nanoparticles produce a cytotoxic effect in lung carcinomas [95]. A high reactivity characterizes the surface of gold nanoparticles. Due to this property, the surface of these nanoparticles can be easily modified or conjugated with functional biomolecules or other materials [35,134]. Gold nanoparticles can be encapsulated in liposomes, conjugated with nucleotides, coated with different polymer layers, or utilized as the core for dendrimers [83]. As mentioned above, nanoparticles are used for the targeted delivery of gene molecules. Of interest is siRNA, which is less stable, and enzymes can be attached to the microenvironment. Nanoparticles have the possibility of altering the fate of siRNA upon in vivo administration [135,136,137]. The advantages of nanoparticles favor siRNA delivery across biological barriers, which can be achieved using different methods: siRNA can be conjugated on the surface of nanoparticles via a gold–thiol bond or electrostatic interactions, or it can adhere to the surface of the nanoparticles using polymer layers [138,139]. Gold nanoparticles are already used as an siRNA carrier system. The most important properties of gold are that it is non-toxic and can form fine nanoparticles, which can be functionalized for efficient gene delivery [34]. Using electrostatic or covalent methods, siRNA can be bound on the surface of the metal. Polyvalent molecules of siRNA can be attached to the surface of gold nanoparticles via thiol groups. These kinds of particles are characterized by a higher stability [139]. If a polyethyleneimine coating is added to the gold nanoparticle, this could render it a perfect siRNA delivery system. The interaction between polyethyleneimine-capped gold nanoparticles and siRNA is electrostatic [140,141]. It is worth mentioning that gold nanoparticles with cationic polymer modifications are excellent gene delivery systems. Gold nanoparticles can become stimuli-responsive, and in this way, siRNA delivery is very efficient [142]. Additionally, researchers have also developed a system represented by a gold nanoparticle-based sensor capable of detecting lung cancer by analyzing the exhaled breath of the patient. Gold nanoparticles were tested as sensors and are capable of detecting lung cancer due to their histology. As sensors, they were capable of distinguishing between the subtypes of lung cancer [139,140,141,142,143,144,145].

Concerning pulmonary cancer management, gold nanoparticles have at least three important advantages. Firstly, gold nanomaterials can be used as a diagnostic tool, offering important advantages in comparison with traditional organic dyes, such as a minimal toxicity and insignificant quenching [146]. Finally, gold nanomaterials exhibit therapeutic effects *per se* due to their implications and use in Photodynamic therapies (PDTs), which have been studied extensively in the chapter on the therapeutic effects of nanomaterials in the current article [147].

Carbon nanotubes are nanosized, hollow, and graphite sheets that are rolled up into a tubular form and belong to the family of fullerenes. These structures are called single-walled carbon nanotubes, if characterized by the presence of a single graphene sheet, or multi-walled carbon nanotubes, if they are formed from several concentric graphene sheets [148]. The diameter of single-walled nanotubes range between 0.5–3 nm, and the length can vary between 20–1000 nm, and as for multi-walled carbon nanotubes, the dimensions are 1.5–100 nm and 1–50 microns, respectively. Single-walled and multi-walled carbon nanotubes can be utilized as nanocarriers for specific drug delivery due to their specific physicochemical and biological characteristics [148,149,150]. Some of these characteristics may include a nanoneedle shape, hollow monolithic structure, high mechanical strength, high electrical and thermal conductivities, and also the ability to make surface adjustments [66]. The main disadvantage of carbon nanotubes as a drug nanocarrier is the poor water solubility and toxicity. The functionalization of carbon nanotubes is an essential key parameter in reducing the toxicity and maximizing the bioavailability of anticancer drugs, and carbon nanotubes are becoming an ideal nanocarrier for cancer therapy [66,151]. These nanostructures were intensively studied in recent years as a nanocarrier for anticancer drug delivery. There are many applications in which carbon nanotubes are very useful, such as gene delivery. The capacity of carbon nanotubes to transport DNA across the cell membrane is widely used in studies that involve gene therapy or gene silencing. A highly selective therapy is needed for cancer therapy, wherein tumor cells will be selectively modulated, so in this case, gene silencing may be performed using siRNA. However, delivering siRNA to specific cells is very problematic, given the instability of siRNA and their low uptake efficiency [21,47,48,49,60,69].

On the other hand, a crucial advantage of using these nano-sized materials in lung cancer treatment is their ability to enhance the effectiveness of chemotherapy, just by their plain administration in combination with such conventional anti-tumoral drugs. In addition, it was shown that using carbon nanotubes may prove to be effective in treating multidrug-resistant and/or radioresistant tumors, a fact that represents another important benefit of these materials [54]. Several studies involving Gemcitabine, Curcumin, Paclitaxel, and DOX carried by carbon nanotubes demonstrated the great versatility of these inorganic materials in the context of their use as drug nanocarriers [152].

### 3.3. siRNA Delivery Systems

RNA interference was first discovered in plants in 2010, and later, the first small interfering delivery nanoparticle was created for effective use in humans. RNA interference is a defense mechanism, helping the eukaryotic cells to destroy the exogenous genes [153,154]. The double-stranded RNA enters the cell and is cleaved in short double-stranded fragments by the Dicer enzyme. Then, each double-stranded siRNA is split between the passenger and guide strands. The passenger strand is degraded, and the guide strand is incorporated into the RNA-induced silencing complex. The guide strand and the complementary sequence in mRNA lead to post-transcriptional gene silencing [143,155,156].

The inhibition of cellular pathways can be achieved with the help of siRNA. Serene can destroy specific mRNA molecules and down-regulate the expression of many multidrug-resistant genes [157]. 

siRNA can target a multitude of undruggable genes, with kinases being the ones that have been validated for traditional small molecule drugs. In cancer, for example, genes are deregulated by high-level amplifications [158,159]. This kind of gene is of interest as a potential therapeutic target. Cancers are initially sensitive to chemotherapy and often adapt tolerance to targeted therapy by gene mutations [160]. siRNA-based drug delivery is appealing, as it can target any mRNA of interest, and signs of progress have been shown for the development of siRNA-based drugs. There are many clinical trials regarding siRNA-based medicines that target the vascular endothelial growth factor (VEGF) pathway [161,162,163]. Researchers have developed different vectors to improve RNA interference therapy in vivo, such as viral vectors, like the adenovirus, or non-viral vectors, which are seemingly the safer alternative. The principal characteristics of non-viral vectors should be their biocompatibility, intracellular uptake, specificity, and better half-life within the bloodstream [164]. Many nanocarriers can be functionalized with different types of nanoparticles. Nanocarriers enter into the specific target cells and act through cellular pathways to deliver siRNA into the cytoplasm. Via endocytosis, nanocarriers are taken up by the cells. Endocytosis is not suitable for all nanocarriers, especially those containing drugs susceptible to lysosomal degradation [66,165]. Many strategies can be used to assist nanocarriers in escaping from degradation. For example, one of these is represented by the flip-flop mechanism. Scientists developed polyelectrolyte complex micelles that can be used as delivery systems for siRNA to silence the VEGF gene in cancer cells [166,167].

The local administration of siRNA is an efficient and convenient method due to the prevention of systemic toxicity [168]. The release of siRNA into the microenvironment of the cells or tissues transforms the siRNA into a biocompatible matrix, which is essential. Regarding lung cancer therapy, this delivery method has a critical role, because the therapeutic agent is transported to the bronchial airways, efficiently targeting the immune cells. The therapeutic potential of siRNA is validated for use within in vivo applications. Though already mentioned, it should be repeatedly stressed that this delivery system has to be characterized by biocompatibility, biodegradability, and non-immunogenicity [148,156].

## 4. Nanocarriers Suitable for Lung Cancer Treatment

Starting from the organic solid lipid nanoparticles (SLNs) to the inorganic nanotubes, the last couple of decades came with indubitably revolutionary drug delivery methods. With significant advantages over conventional therapies, nanocarriers promise to solve a great number of issues in the contemporaneous medical world [169]. Currently, with the great majority of the FDA cancer nanotherapy solutions being based on liposomal formulations (8 out of 12) (Anon n.d.), there is still a lot of room for discoveries regarding this matter. In addition, considering that the treatment suggestions and choices of the American Cancer Society for non-small cell lung cancer do not include nanotechnology-based therapies at all at this time, this is a clear sign that medical society is still not completely embracing these alternatives [170].

One might find it interesting that even the most recent reference reviews [171,172] on the specific theme of nanocarriers suitable for lung cancer treatment recognize the great number of doubts and burdens that are still to be considered in this niched research area. At the moment, only a few experiments have apparently been conducted with the specific aim of targeting lung cancer via nanoparticles, with the great majority of them focusing rather on the usage of these nano-sized carriers in oncology therapies [173]. However, clear paths and perspectives are already shaped and offer important glimpses of hope for the future. Several nanocarriers that were already largely studied for targeting lung cancer are presented in Table 2, along with their characteristics.

Far from being exhaustive, Table 2 engulfs a mixture of already well-established, conventional nano-sized carriers and novel, intriguing delivery agents, which may someday be vital to the targeted therapy of lung cancer. With inhalation playing an expected crucial role in this non-systemic drug delivery approach, several prospective nanocarriers seem to be potential serious candidates in this race for a specific, non-invasive anti-tumoral therapy [185]. However, judging by the FDA decisions made so far, for the moment, it may be advisable that research should concentrate more on liposomal formulations alone. Apart from having well-known advantages over conventional therapies [186], liposome-based nanocarriers are indubitably versatile platforms, supporting a wide range of coatings and different types of loads [187].

## 5. Therapeutic Effects of Nanomaterials

The impressive versatility of nanomaterials is not solely based on their ability to deliver various compounds or genes in different dosages at specifically targeted sites [188]. While research efforts were mainly channeled in this direction in the last decades, nano-sized materials can be regarded alone as valuable therapeutic agents. One curious example was already presented in the section on non-polymeric particles, where we mentioned the case of plain carbon nanotubes used as tools for chemotherapy potentiation. This effect may be due to the possible long-term immunostimulatory effects of the nanotubes, which was also observed in a similar study [54]. In this section, we will describe two of the most intensely studied techniques that use nanoparticles as therapeutic agents or smart integrative nanoplatforms, rather than simple drug carriers.

### 5.1. Photothermal Therapy (PTT)

Already known for more than a couple of decays, one of the most intensely studied procedures involving nanosystems as active curing instruments is photothermal therapy (PTT). In simple terms, this method relies on the cancer cell lysis caused by the high temperature achieved in the tumoral tissue by exposure to near-infrared (NIR) light. The crucial role of the nanoagents in this operation is, evidently, to enhance the selectivity of heat production at the lesional site [21]. By using nano-sized particles as NIR absorbents, the efficiency of the heat production in the tumoral microenvironment is significantly greater, and the lesional effect on the circumambient normal tissue would be minimized, not to mention the avoidance of unwanted systemic side effects [189].

Gold nanoshells were the very first such NIR absorbents used in PTT, with an evidence-based effectiveness. Developed in the mid-1990s as PEGylated silica-cored Au nanoshells, they later appeared in 2008 as absorbent agents for the AuroLase^®^ Therapy (Nanospectra Biosciences, Houston, TX, USA) [190]. The preclinical studies confirmed both the accumulation of these particles at the tumoral site and their effectiveness as light-to-heat conversion mediators. However, according to Nanospectra Biosciences, the proprietor of this technology, the nanoshells are currently only available for ‘designated FDA sanctioned clinical studies’. Two clinical trials are being conducted at the moment to further investigate the safety and efficiency of these NIR absorbents [191].

Lately, materials such as semiconductors, graphene nanoparticles, polypyrrole nanoparticles, copper sulphide nanocrystals, and others are starting to be considered as possible alternatives as nano-sized light absorbents to noble metals [192]. To avoid diversion from our main subject, we recommend the study of two comprehensive reviews on this matter, which best summarize the current aspects of nanomaterials used in PTT procedures.

### 5.2. Photodynamic Therapy (PDT)

Another emerging therapeutic solution that uses plain nanomaterials is Photodynamic therapy (PDT). The mechanism of action is already relatively well known, consisting basically of a photosensitizing (PS) agent being activated by light of a specific wavelength. After the photons activate the respective sensor, this will produce reactive singlet oxygen, which is largely known for its cellular cytotoxic effects. Using nano-sized materials as photosensitive agents for PDT implementation in cancer therapy would be a logical move to potentiate the specificity of this technique [193].

Interestingly, combining the use of nanoparticles and the PDT method encouraged the already known phenomena, called theranostics. This brand new concept suggests a synergy between diagnostics and therapy, a strategy that was proven to be easily achieved using nano-sized particles as PSs carriers in PTD [194].

Such an example would be the utilization of poly(vinyl alcohol)-porphyrin nanoparticles (PPNs). Specifically, those carriers function as PSs and are also able to transport antitumoral drugs (such as DOX-tested drugs in the cited experiment), which would be released at the tumoral site, once the PPNs are activated by NIR light. Not only did these smart nanoplatforms release active agents at the specific tumoral site, but they also combined PTT and PDT techniques to finally achieve a 100% survival rate in mice after 45 days of close observation and treatment. In addition, only one in six mice developed recurrent tumors [195]. Finally, a precision of approximately 95% was reported for these nanoparticles used as imaging tools, which may be involved in tumoral diagnostics and monitoring. Another interesting approach suggests the combination of inorganic materials using the PDT technique. Porphyrin-silica nanoparticles may be such an example, which proved to be useful due to both intense their fluorescence (that may be suitable for cell labelling) and sufficient reactive oxygen species (ROS) generation to inhibit tumor growth [196].

## 6. Biocompatibility

Nanoparticles and nanomaterials have increasingly found practical applications in several fields and possess the capacity to change the methods of diagnostics or therapeutics currently in use [24,197]. Biocompatibility refers to the ability of a biomaterial to perform its desired function with respect to medical therapy, without eliciting any undesirable local or systemic effects in the recipient or beneficiary of that therapy, while generating the most suitable beneficial cellular or tissue response in that specific situation and optimizing the clinically relevant performance of that therapy [198]. Nanoparticles should be analyzed before they are approved for use in biomedical applications, such as the treatment of different types of cancer. For such applications, nanoparticles or nanomaterials should be tested on various tissular or cellular types to evaluate the negative and positive effects on the human body [47,199]. Nanotechnology-based delivery systems have to be improved to increase their bioavailability, biocompatibility, and safety profiles to take advantage of the impressive potential of these in varied biomedical applications, including anti-tumor activity or gene therapy [32]. Concerning biomedical applications, different types of nanoparticles may enter the body and contact tissues and cells directly, making it necessary to fully explore their biocompatibility, since neither their effect on all tissue or cell types nor all their interactions are completely understood [200]. As of now, cell cultures are very convenient for understanding the biological effects of the activities of nanoparticles and nanomaterials, their toxicity, and their action mechanism [18,201].

## 7. Conclusions

Nanotechnology is a rapidly progressing area of science and offers a chance to change and to develop characteristics that are relevant for applications in diagnosis and new strategies for improving properties that are relevant for applications in drug delivery. While nanotechnology is still at an early stage of its evolution, several drugs that utilize nanotechnology have been approved, while many others are being studied that have a high potential to offer safer, more effective, and even personalized treatments. Liposomes are defined by a unique structure that is similar to cellular membranes, and they are considered to be more biocompatible than other synthetic materials. These characteristics make them highly valuable for drug transport systems, and they are being developed as nanocarriers. The structural properties of dendrimers and the fact that they can be almost precisely controllable support their utilization in the delivery field in cancer research. Polymers are a great candidate for administering the medication via the oral, intravenous, or a combined route because of their advantages: biocompatibility, biodegradability, and lower toxicity. Another innovative drug delivery system is represented by micelles due to their stability in physiological conditions or high accumulation of drugs at the target site. Inorganic materials, such as gold nanoparticles, offer a wide variety of attributes that allow them to be adapted to either provide or enhance diagnosis. Due to the investigations made, it should also be mentioned that nanotubes may be employed in the diagnosis of certain disorders. The local administration or release of siRNA into the cellular or tissular microenvironment transforms the siRNA into a biocompatible matrix. By discovering new nanosystems that are involved in cancer signaling pathways, a great opportunity arises to ultimately identify a personalized therapy that is effective for each patient.

## Figures and Tables

**Table 1:** Nanosystems: principal categories, characteristics, and applications.

Major Categories of Nanosystems	Types of Nanocarriers	Characteristics
	Carbon nanotubes	Size: single-walled carbon nanotubes (0.5–1.5 nm) multi-walled carbon nanotubes (>100 nm) Remarkable strength, unique electrical properties, functionalization-enhanced solubility, penetration to cell cytoplasm and to the nucleus, as a carrier for gene delivery [27,28]
	Dendrimers	Size: <10 nm Highly branched, nearly monodisperse polymer system, controlled delivery of bio-actives [29,30,31]
	Liposomes	Size: 50–100 nm Phospholipid vesicles, biocompatible, versatile, passive and active delivery of genes, peptides, and proteins [24,32,33]
	Metallic nanoparticles	Size: <100 nm Stable, high surface area available for functionalization, diagnostic value, drug and gene therapy [34,35]
	Nanocrystals (Quantum dots)	Size: 2–9.5 nm Semiconducting material, VDNA hybridization, immunoassay [36,37]
	Micelles	Size: 10–100 nm High drug entrapment, biostability, passive and active drugs transport [38,39]
	Nanoparticles	Size: 10–1000 nm Biodegradable, biocompatible, complete drug protection, excellent carrier for drugs, passive and active drugs transport [40]

**Table 2:** A succinct list comprising the most interesting, promising, and already tested nanocarriers used in lung cancer therapy experiments.

Nanocarrier	Carrier Material and Characteristics
**Tecemotide**	Carrier material: Synthetic lipopeptide Found under the form of liposomes, actively targets MUC1 tumor-associated antigen (TAA), aberrantly expressed by over 90% in lung cancers, well-tolerated by the human body, but low or imperceptible efficacy [174,175]
**ExtraCRAd**	Carrier material: Biohybrid viral nanoparticle Consists of an oncolytic virus artificially encapsulated in tumor cancer membranes carrying tumor antigens, preliminary results involving it showed indisputable tumor control in murine models of lung cancer and melanoma [176]
**HVJ-E**	Carrier material: Viral envelope Represents the hemagglutinating virus of Japan envelope (HJ-E) obtained from the replication-deficient Sendai virus, actively targets ICAM-1, largely present in lung cancer, very promising drug delivery agent, considering that it already has thoroughly proven direct oncolytic effects, proven to be suitable as a gene delivery system [177,178,179,180]
**Bacterial-derived minicells**	Carrier material: Bacterially derived nano-sized particles Can be loaded with a wide range of various chemotherapeutic agents, coating them with specific, customized antibodies will result in a very high tumoral specificity, showed enhanced biodistribution to the lungs, while carrying doxycycline [181,182]
**Polymeric nanoparticles**	Carrier material: Polymer-based nanoparticles or lipid-polymer hybrid nanoparticles Particles may be co-decorated with Nitroimidazoles (NI) to improve the targeting of the hypoxic tumoral environment and/or Hyaluronic acid (HA) to improve (lung) tumoral targeting even more by binding to the CD44 cell marker, and such carriers loaded with cisplatin showed an impressive tolerability and promising results for prospective lung cancer treatment [183,184]

## Data Availability

Not applicable.
